# The Book World of Medicine and Science

**Published:** 1906-11-24

**Authors:** 


### The Book World of Medicine and Science.

Lectures on Diseases of the Lungs. By J. A. Lindsay, M.D., F.R.C.P. (Lond.), Professor of Medicine, Queen's College, Belfast. Second edition, enlarged and re-written. (London : Baillière, Tindall, and Cox. Pp. 509. Demy 8vo. 1906. Price 10s. 6d. net.)

We welcome a second edition of Professor Lindsay's volume of clinical lectures, which has been considerably altered by the omission of the portion dealing with diseases of the heart, the space so gained being devoted to pulmonary affections. The first seven lectures are concerned with diagnosis, and present an excellent statement of the methods of investigation in diseases of the chest. For these alone the book would have been valuable, for not only does the author collate and harmonise the confusing terminology of the leading writers on physical examination, but his criticisms express the opinions of a skilled clinician and one more concerned with actualities than the niceties of text-book classification. These, as well as the remaining lectures, are distinguished by clear and graphic description and are eminently practical in character, the problems presented in actually dealing with patients being frankly stated and carefully considered. It would be invidious to particularise, but the lecture on the conditions which simulate phthisis deserves the attention of every practitioner. The prognosis of pulmonary tuberculosis is very fully considered, and the difficulty of choosing a suitable health resort for a particular patient should be greatly simplified by a study of the indications given under a number of well-chosen clinical types. The author takes a distinctly unfavourable view of the employment of tuberculin, and it is perhaps owing to this that the section dealing with the specific treatment of pulmonary tuberculosis strikes us as somewhat meagre. The theory of opsonins and the possibility of increasing the opsonic power of the blood in phthiscal patients by the injection of tuberculin is mentioned, but the importance of avoiding the repetition of the injection during the "negative phase" seems scarcely to have been sufficiently appreciated. The volume is well printed and is provided with a copious and efficient index.

Bodily Deformities. By the late E. J. Chance, F.R.C.S. Edited by John Poland, F.R.C.S. Second Edition. (London : Smith, Elder. 1905. Pp. xlviii+315. Illustrations 121. Price 6s. net.)

After more than forty years a second edition has appeared of the first volume of Mr. Chance's book on Bodily Deformities. The second volume exists in manuscript and is to be published by Mr. Poland, who has re-edited and revised the first edition. The editor has wisely put his annotations in footnotes, leaving the text of the book practically unaltered to stand as a monument to the anatomical and physiological learning and painstaking research of the author. The subject matter of the book is necessarily not new, but the book is of value for its historical interest. The origin and causes of congenital and acquired deformities are exhaustively dealt with in a manner which is an education in the conduction of research. The introduction is not to be skipped, for it is full of interesting details of the beginnings of orthopædic surgery, when Abernethy sent his cases of club-foot to an instrument-maker to see if he could devise a method of treatment, and Cheselden admitted that the first knowledge he had of the cure of club-foot was from Mr. Presgrove, a professed bone-setter, down to the introduction of tenotomy and the foundation of the scientific treatment of deformities as a branch of legitimate medicine. There is food for much reflection in the book, for many of the difficulties which were recognised by Chance still beset the path of the orthopædic surgeon. Treatment has unfortunately little part in the present volume, but we hope Mr. Poland will shortly publish the second volume, and so complete the record of Mr. Chance's contributions to an important branch of surgery.

Problems in Animal Metabolism. By J. B. Leather. (London : John Murray).

This book deals with intricate subjects in a lucid interesting way. The chemistry of physiology has taxed the energy and acumen of several great scientific workers of the past century, yet problems which presented themselves to those workers have not been fully solved by the many investigators who have followed in their footsteps. How sugar, such an important element of nutrition, is formed within the body, and how it is broken down are still vexed questions, and in what way proteids nourish us, and why the nitrogen they contain should be an essential element of our food require further elucidation. Uric acid, too, so familiar by name we may almost say to the public at large, is still to a considerable extent veiled in mystery. If, however, conclusions upon important points have not been arrived at, the amount of knowledge that has been gained concerning the various chemical products of animal activity is very great. When it is said that in the sprouting seedling of a plant the proteid nutriment breaks up into leucine, iso-leucine, amido-valerianic acid, alanine glutannic acid, aspartic acid, phenyl-alanine, tyrosine, pyrrholidine carboxylic acid, cystine, tryptophane, lysine, arginine, and histidine, to be built up again during growth into the proteids of the plant, it will be understood that if chemical action is so complicated in low forms of life the unravelling of the mysteries of metabolism of the human body may well occupy many studious workers for years still to come. Amongst the items of knowledge of comparatively recent date, which are obviously of practical character, it may be mentioned that fat can be used for muscular work no less economically than either proteids or carbohydrates. Another item of interest in connection with fat, which is perhaps more pathological than physiological, is that so-called fatty degeneration does not in reality occur. In certain conditions fat globules are stored in the heart-muscle or liver cells, but are not formed in the muscle or liver cells.

Food for the Sick. By Mary Truman, M.R.B.N.A., and Edith Sykes, A.R.S.I., authors of "Nursing Old Age," etc. Third edition, 15,000th. (London : Allman and Son, Ltd. 3d.)

This is a very compendious and useful work, containing many recipes for the preparation of food for the sick. The authors are evidently well experienced, and the recipes are of a high-class and useful character. The nominal price brings it within the reach of the housewife.

